# Contextual Similarity Between Successive Targets Modulates Inhibition of Return in the Target-Target Paradigm

**DOI:** 10.3389/fpsyg.2020.02052

**Published:** 2020-09-08

**Authors:** Hsuan-Fu Chao, Chun-Yu Kuo, Makayla S. Chen, Fei-Shan Hsiao

**Affiliations:** ^1^Department of Psychology, Chung Yuan Christian University, Taoyuan City, Taiwan; ^2^Department of Adult & Continuing Education, National Taiwan Normal University, Taipei City, Taiwan; ^3^School of Psychology, Speech and Hearing, University of Canterbury, Christchurch, New Zealand

**Keywords:** contextual similarity, cuing, inhibition of return, memory retrieval, response time distribution

## Abstract

Inhibition of return (IOR) refers to slower responses to a target presented at a previously cued vs. uncued location. The present study investigated the role of memory retrieval in IOR by manipulating the contextual similarity between two successive targets in the target-target IOR paradigm. Successive targets were presented in either the same color (same-context condition) or different colors (different-context condition). Results of two experiments showed that IOR was greater in the same-context than the different-context condition. In addition, Experiment 2 showed that this context effect occurs with long response times (RTs), suggesting that memory retrieval, which requires time to manifest, plays an important role in IOR.

## Introduction

Inhibition of return (IOR) is the delayed response to a target presented at a previously cued location (Posner and Cohen, 1984; Posner et al., 1985; see Klein, 2000, for a review). IOR is often observed in cuing paradigms. After the presentation of an abrupt-onset cue, a target is presented at either the location previously occupied by the cue (cued condition) or a different, uncued location (uncued condition). Compared to the uncued condition, responses to targets at the cued location are usually faster, when the cue-to-target onset asynchrony (CTOA) interval is short, and slower (IOR), when the CTOA interval is long. Posner and colleagues (Posner and Cohen, 1984; Posner et al., 1985) proposed that IOR is the result of attentional inhibition of previously attended spatial locations.

Although IOR is usually viewed as an attentional phenomenon, recent studies suggest that it may involve memory retrieval. This is supported by evidence of long-term IOR (Tipper et al., 2003; Kessler and Tipper, 2004; Grison et al., 2005; Wilson et al., 2006). Tipper et al. (2003) proposed that transient attentional inhibitory effects are encoded into memory representations. After a long temporal interval, these representations can be retrieved and the inhibitory effect reinstated when appropriate retrieval cues are present. To support this proposal, they demonstrated that when a cued face was presented again after 3 or 13 min, responses to targets presented in that face were slower than when targets were presented in an uncued face. Because the delay was much longer than in standard short-term IOR effects, they reasoned that slower responses in the cued condition reflected retrieval of previously encoded inhibitory effects from long-term memory (see Kessler and Tipper, 2004; Grison et al., 2005, for similar findings). Wilson et al. (2006) further demonstrated that long-term IOR could be obtained for spatial locations, suggesting the importance of memory retrieval. Unlike Tipper et al. (2003) and Wilson et al. (2006) suggested that the response associated with the stimulus, rather than the inhibitory state, is encoded into long-term memory and produces long-term IOR effects when retrieved.

The demonstration of memory retrieval in long-term IOR prompts an intriguing question: what is the role of memory retrieval in short-term IOR? Wilson et al. (2006) suggested that the same retrieval mechanism might be responsible for both short– and long-term IOR. In other words, memory retrieval may be a key mechanism underlying short-term IOR. Nevertheless, as Wilson et al. (2006) noted, this requires further investigation. While memory retrieval in long-term IOR is supported by long temporal intervals between the cue and target (in which case the transient inhibitory state should be absent), the evidence for memory retrieval in short-term IOR is less clear.

One way to investigate the role of memory retrieval in short-term IOR is to study the influence of contextual similarity on IOR. According to the encoding specificity principle of Tulving and Thomson (1973), the contextual similarity between memory encoding and retrieval modulates retrieval of previous memory episodes. Consequently, the contextual similarity effect has been extensively employed as a memory retrieval index in the literature on negative priming (NP), which is an aftereffect of selective attention (e.g., Neill, 1997; Fox and de Fockert, 1998; Stolz and Neely, 2001; Chao, 2009; Tse et al., 2011). Similarly, in the present study, the impact of contextual similarity on IOR should be investigated.

The target-target paradigm was used in the present study to investigate the effect of contextual similarity between successive targets on IOR. IOR can be obtained in a target-target paradigm that involves responding to a target presented at either the same or different locations in two successive trials (e.g., Maylor and Hockey, 1985; Tanaka and Shimojo, 1996; Pratt and Castel, 2001; Betta et al., 2007). The benefit of using this paradigm is that contextual similarity can be manipulated by simply varying the color of successive targets. When two successive targets are presented in the same color, they share the same color context (same-context condition); when two successive targets are presented in different colors, they are associated with different color contexts (different-context condition). Hence, the focus of the present study would be the interaction between cue (location repetition) and context (color repetition). Previously, addressing a different research question – does color-based IOR exist or not? – Kwak and Egeth (1992) conducted a similar experiment in which color repetition and location repetition were also manipulated. They observed an interaction between location and color repetition but questioned the reliability of the effect of color repetition. Law et al. (1995) suggested that it is important to include a neutral distractor and observed a reliable effect of color-based IOR. However, Law et al. did not manipulate location repetition and, therefore, did not examine the interaction between location repetition and color repetition. Hence, the design of the present study was a combination of Kwak and Egeth (1992) and Law et al. (1995): the neutral distractor was presented at the beginning of each trial while both location repetition and color repetition were manipulated. The present study expected that if IOR involves context-dependent memory retrieval (e.g., Tulving and Thomson, 1973), IOR should be greater in the same– than the different-context condition because it would be easier to retrieve the memory episode for the previous target when the context was identical (i.e., the target was in the same color) than when the context was different (i.e., the target was a different color). By contrast, if IOR does not involve a memory retrieval process, IOR should not be affected by the manipulation of retrieval context.

In addition, in order to further examine the role of the context-dependence of memory retrieval, the present study investigated whether the impact of retrieval context on IOR is modulated by the time course of the processing of the target event. In their study of the contextual similarity effect in NP, Tse et al. (2011) proposed that the contextual similarity effect is easier to observe with slower rather than quicker responses. To evaluate this proposal, they sorted the data in each condition from quickest to slowest. They then divided the data in each condition into eight smaller proportions and calculated the mean of each of the eight proportions within each condition. Consistent with their proposal, they found that the contextual similarity effect in NP was larger in the longer RT bins than in the shorter RT bins. If the contextual similarity effect in IOR is governed by a memory retrieval mechanism with similar characteristics, then the contextual similarity effect should be greater in the longer RT bins as well. Hence, we further analyzed the vincentized cumulative RT distribution (Ratcliff, 1979) to explore the relationship between the contextual similarity effect in IOR and RT bins.

## Experiment 1

### Materials and Methods

#### Participants

Forty-two undergraduate students participated in this experiment at Chung Yuan Christian University and received NT$240 or NT$300 as compensation. Participants had normal or corrected-to-normal vision. All participants read and signed an informed consent form at the beginning of the experiment.

#### Stimuli

Three boxes were presented in white as placeholders. At a viewing distance of approximately 57 cm, each box subtended a width of 2.8° and a height of 2.0°, with a border thickness of 0.1°. The three boxes were presented at the center of the screen, aligned on the horizontal meridian. The center-to-center distance between adjacent boxes was 4.7°. The central box was cued at the beginning of each trial by increasing the width of that box outline to 0.3°. The target was a dot presented in the center of either the left or right box. The color of target was either red or green.

#### Procedure

The experiment was administered using DMDX software (Forster and Forster, 2003). To manipulate color context between successive targets, couplets of two successive trials were used. In the test couplets, there were targets in both the first and second trials. The target in the first trial served as the cue, and performance in the second trial could reveal an IOR effect. The targets in the first and second trials were presented in either the same or different colors. In the catch couplets, there was a target in the first trial and no target in the second trial, no target in the first trial and a target in the second trial, or no target in either trial.

Before the beginning of the experiment, informed consent was obtained from each participant. There was a practice block of eight test couplets and five catch couplets at the beginning of the experiment, followed by 128 test couplets and 48 catch couplets. Half of the test couplets were cued, such that targets were presented at the same location; the other half were uncued, such that targets were presented at different locations. Half of the test couplets were same-context couplets and the remainder were different-context couplets. Each color was presented with equal frequency.

**Figure 1:**
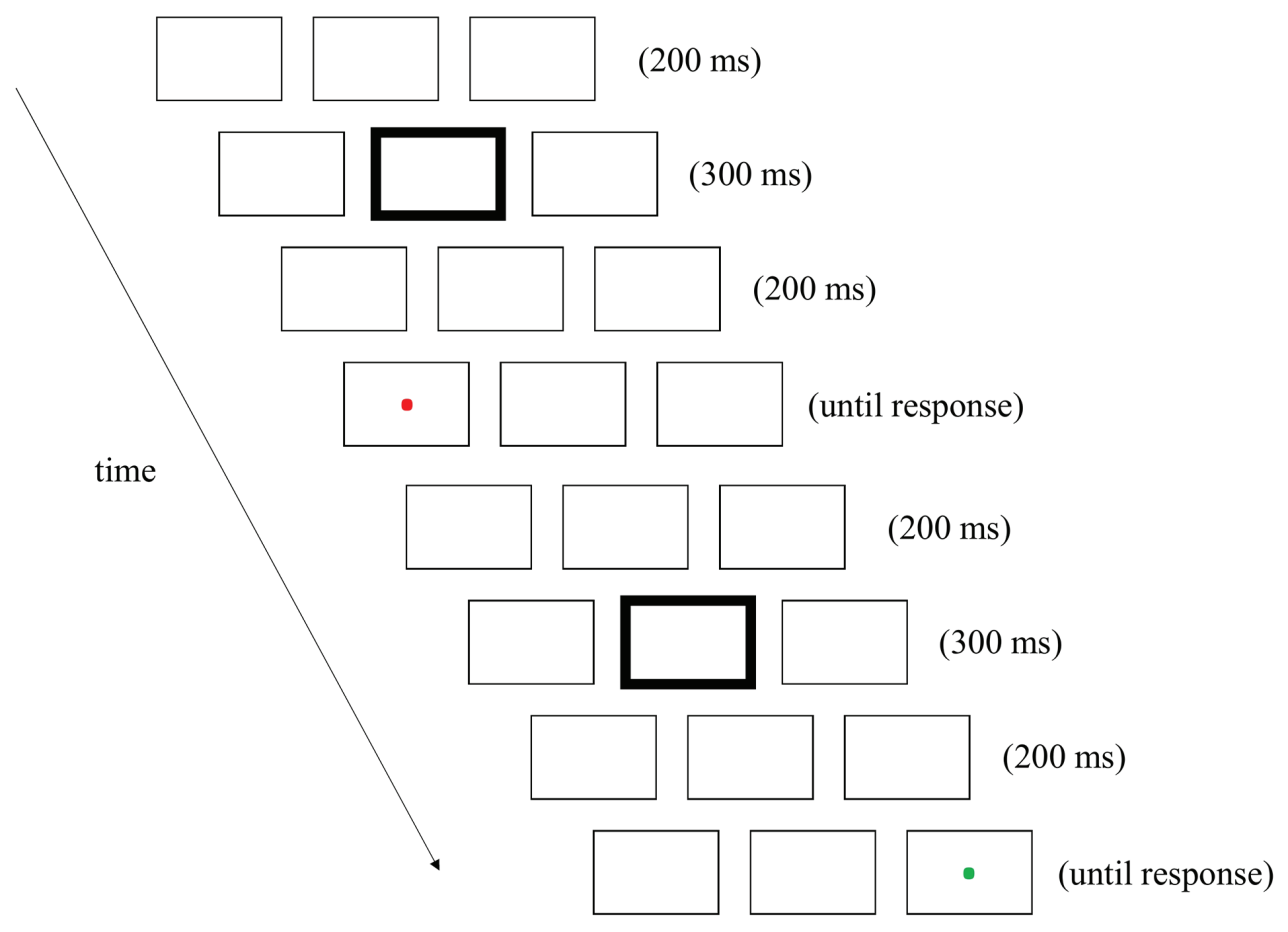
An example of a couplet in Experiment 1 (not to scale). There were two trials in each couplet. Each trial began with the display of the three placeholders. At the beginning of each trial, a 300-ms fixation cue was presented in the central box by increasing the width of that box outline. After the offset of the fixation cue, the placeholders were presented on the screen for 200 ms. A target, a red or green probing dot, was then presented in either the left or right box, or no target was presented.

This experiment began with the display of the three placeholders. These placeholders were presented on each trial and during the intervals between successive trials. There were two trials in a couplet (see Figure 1 for an example). At the beginning of each trial, a 300-ms fixation cue was presented in the central box. The location and color of the fixation cue were different from the targets and could therefore serve as a neutral distractor. After cue offset, the placeholders were presented on the screen for another 200 ms. A target was then presented in either the left or right box, or no target was presented. Participants were instructed to press the left button of the mouse when they saw a dot, regardless of its color. The interval between successive trials was 200 ms. There were rest breaks after each block of 44 couplets.

### Results

The response to the target in the second trial of each test couplet was only analyzed if the response to the target in the first trial of the couplet was correct. In total, 0.9% of the data were excluded because of errors in responding to the target in the first trial. In addition, outliers were excluded based on the non-recursive method criterion (Van Selst and Jolicoeur, 1994). In total, 3.1% of the data were excluded as outliers.

#### Analysis of the Overall Data

Table 1 shows the average correct RTs and error rates in each condition. Both the RT and error rate data were analyzed by a 2 (cue: valid/invalid) × 2 (context: same/different) repeated-measures analysis of variance (ANOVA).

**Table 1:** Average correct response times (ms) and error rates (%, in parentheses) as a function of context and cue in Experiment 1.

Cue	Same context	Different context
Valid	287 (0.7)	285 (0.5)
Invalid	264 (0.5)	272 (0.6)
IOR	−23 (−0.2)	−13 (0.1)

IOR (inhibition of return) = Invalid − Valid.

Analysis of the RT data showed a significant main effect of cue, *F*(1,41) = 30.95, *p* < 0.0001, $\eta_{p}^{2}$ = 0.43, indicating a significant IOR effect. The main effect of context was not significant, *F*(1,41) = 0.98, *p* > 0.20, $\eta_{p}^{2}$ = 0.02. More importantly, the interaction between cue and context was significant, *F*(1, 41) = 5.05, *p* < 0.05, $\eta_{p}^{2}$ = 0.11, indicating that the IOR effect was larger when context was the same [−24 ms; *F*(1,82) = 34.21, *p* < 0.0001, $\eta_{p}^{2}$ = 0.29] vs. different [−13 ms; *F*(1,82) = 10.76, *p* < 0.01, $\eta_{p}^{2}$ = 0.12]. Finally, analysis of error rates revealed no significant effects, *p*s > 0.20.

#### Analysis of the Cuing Effect as a Function of RT

**Figure 2:**
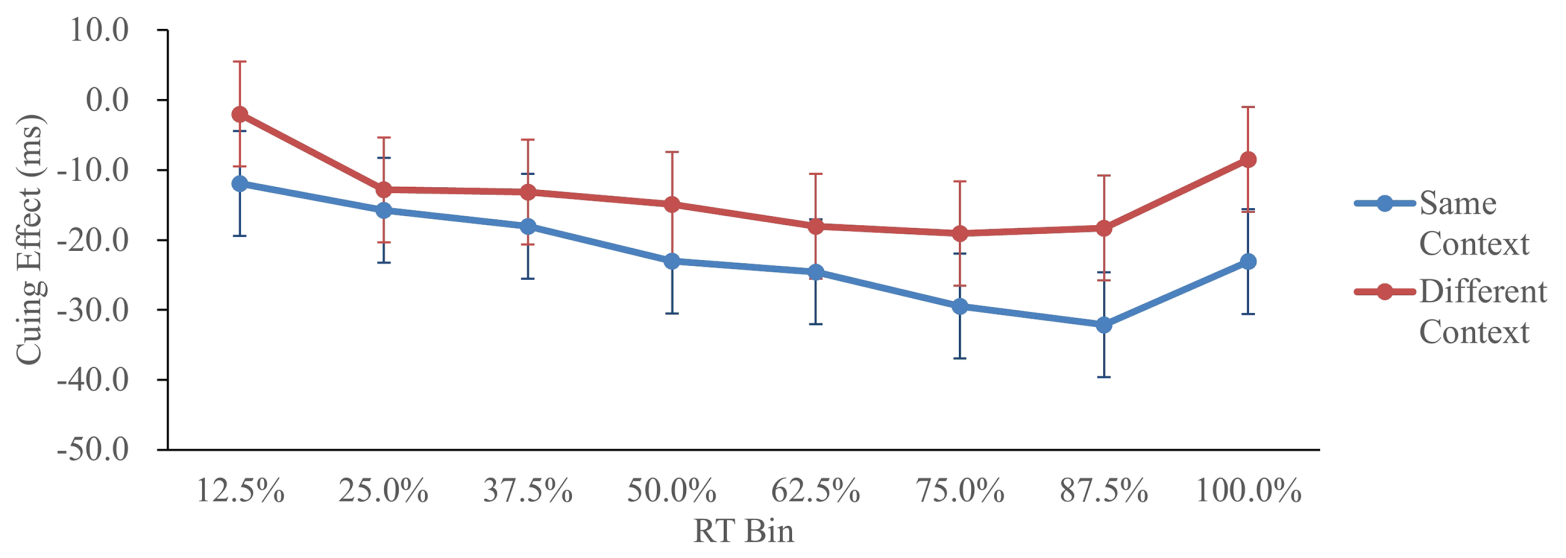
Cuing effect in Experiment 1, as a function of context and response time (RT) bin. Cuing effect = invalid RT − valid RT. Error bars represent the 95% confidence interval, based on the method of Jarmasz and Hollands (2009).

Figure 2 shows the cuing effect (invalid RT−valid RT) in each condition as a function of RT distribution, similar to the procedure of Tse et al. (2011).

The cuing effects were analyzed by a 2 (context: same/different) × 8 (RT bin: 12.5/25/37.5/50/62.5/75/87.5/100%) repeated-measures ANOVA. The main effect of context was significant, *F*(1,41) = 4.44, *p* < 0.05, $\eta_{p}^{2}$ = 0.10, showing larger IOR in the same-context condition. The main effect of RT bin was significant, *F*(7,287) = 4.23, *p* < 0.05, $\eta_{p}^{2}$ = 0.09, showing that the IOR effects in the 75, 87.5, and 100% RT bins were larger than that in the 12.5% RT bin (Tukey’s test, *p* < 0.05). That is, IOR was larger when the RTs were longer. Finally, the interaction of context and RT bin did not reach significance, *F*(7,287) = 0.58, *p* > 0.20, $\eta_{p}^{2}$ = 0.01.

### Discussion

The results of Experiment 1 showed that IOR was larger in the same-context condition than in the different-context condition. This finding suggests the role of context-dependent memory retrieval in IOR.

The magnitude of the IOR effect was further analyzed according to the vincentized cumulative RT distribution (Ratcliff, 1979). The results suggest that the IOR effect was larger when the context was the same and when the RTs were longer. However, although it appears that the effect of context on IOR was larger when the RTs were long, the interaction between the context and RT bin was not significant. Hence, the results of Experiment 1 did not provide strong evidence supporting time-dependent memory retrieval for IOR to manifest.

Regarding the effect of color-based IOR, the main effect of color repetition (i.e., context) was not significant. In fact, color repetition led to a benefit rather than a cost in the invalid condition [8 ms; *F*(1,82) = 4.57, *p* < 0.05, $\eta_{p}^{2}$ = 0.05] and a nonsignificant cost in the valid condition [−2 ms; *F*(1,82) = 0.30, *p* > 0.20, $\eta_{p}^{2}$ = 0.00]. Although neutral distractors were included as Law et al. (1995) suggested, the findings of Experiment 1 are consistent with Kwak and Egeth (1992) but inconsistent with Law et al. (1995). Such differences among these studies suggest that color-based IOR may not be stable in certain circumstances.

One potential limitation of Experiment 1 is that the stimulus onset asynchrony (SOA) between the fixation cue and target was fixed at 500 ms. Hence, the time when the target would occur was predictable. To eliminate this limitation, Experiment 2 replicated Experiment 1 with varying SOA.

## Experiment 2

Experiment 2 replicated Experiment 1 while the SOA between the fixation cue and target was manipulated as either 500 or 700 ms. Owing to this manipulation, the SOAs in each couplet could be either the same or different.

In the study of NP by Neill (1997), whether the SOA between the target and distractor in the prime trial and the SOA in the probe trial were the same or different modulated the NP effect. In other words, the SOA could be another source of context. Hence, whether the SOAs were the same or different was further included as a new variable in the present experiment.

### Materials and Methods

#### Participants

Another 60 undergraduate students at Chung Yuan Christian University participated in this experiment and received NT$260 or NT$300 as compensation. Participants had normal or corrected-to-normal vision. All participants read and signed an informed consent form at the beginning of the experiment.

#### Stimuli and Procedure

**Figure 3:**
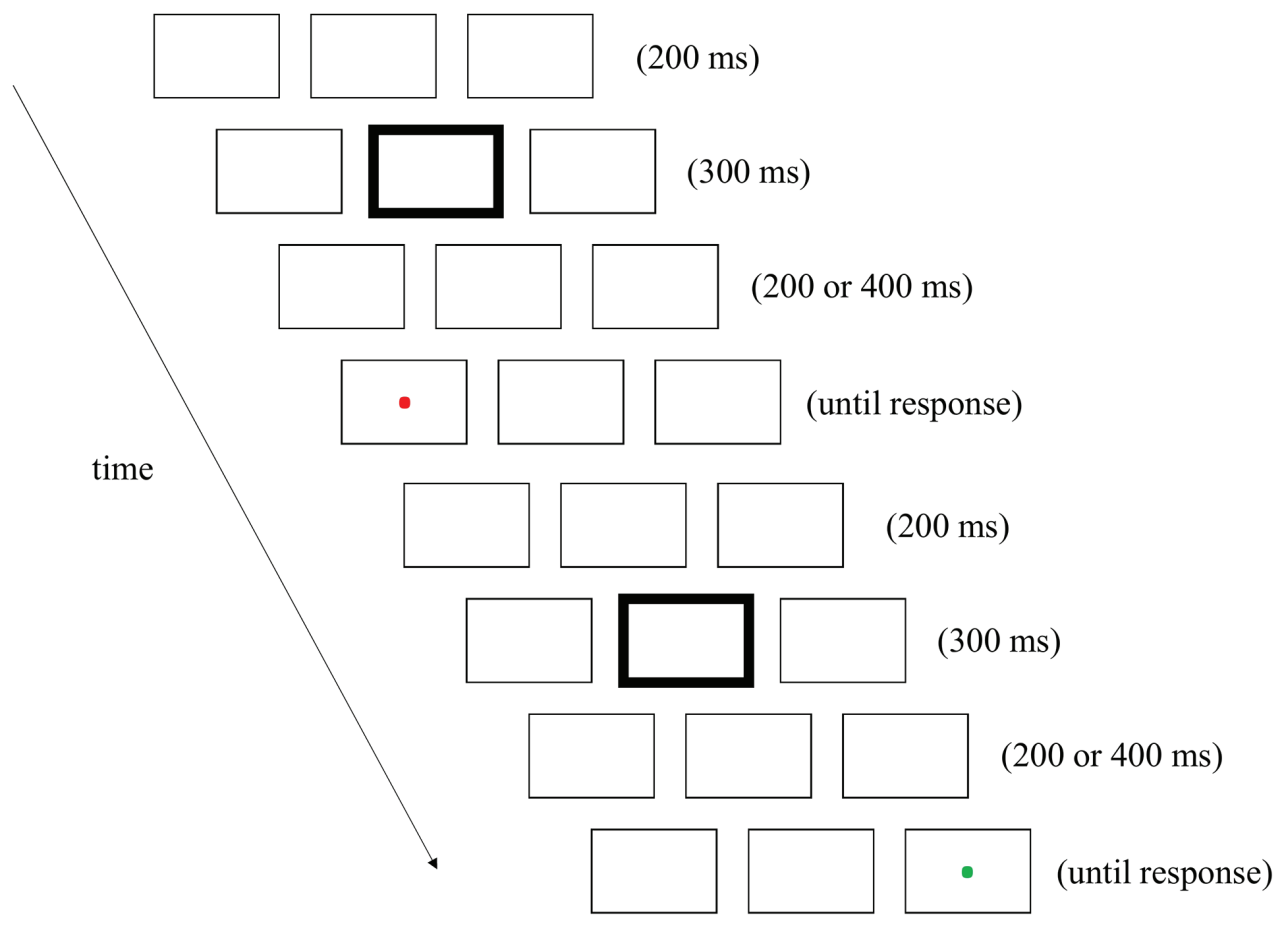
An example of a couplet in Experiment 2. The sequence was similar to that of Experiment 1, with one exception: the interval between the fixation cue and target was either 200 or 400 ms.

The stimuli and procedures of Experiment 2 were identical to those of Experiment 1 with the following exceptions. First, after the offset of the 300-ms fixation cue, the placeholders were presented on the screen for either 200 or 400 ms (see Figure 3 for an example). Second, owing to the inclusion of a new variable, the number of trials was doubled. The SOAs were the same in half of the couplets and different in the remaining couplets.

### Results

Data were excluded based on the criteria in Experiment 1. First, 0.2% of the data were excluded because of errors responding to the target in the first trial. Second, 2.9% of the data were excluded as outliers.

#### Analysis of the Overall Data

Table 2 shows the average correct RTs and error rates in each condition. Both the RT and error rate data were analyzed by a 2 (cue: valid/invalid) × 2 (context: same/different) × 2 (SOA: same/different) repeated-measures ANOVA.

**Table 2:** Average correct response times (ms) and error rates (%, in parentheses) as a function of context, stimulus onset asynchrony (SOA), and cue in Experiment 2.

Cue	Same context		Different context	
	Same SOA	Different SOA	Same SOA	Different SOA
Valid	291 (0.1)	296 (0.1)	287 (0.1)	293 (0.2)
Invalid	271 (0.0)	275 (0.2)	272 (0.1)	276 (0.2)
IOR	−20 (−0.1)	−21 (0.1)	−15 (0.0)	−17 (0.0)

IOR (inhibition of return) = Invalid − Valid.

Analysis of the RT data showed a significant main effect of cue, *F*(1,59) = 188.31, *p* < 0.0001, $\eta_{p}^{2}$ = 0.76, indicating a significant IOR effect. The main effect of context was not significant, *F*(1,59) = 2.71, *p* > 0.10, $\eta_{p}^{2}$ = 0.04. More importantly, the interaction between cue and context was significant, *F*(1,59) = 8.11, *p* < 0.01, $\eta_{p}^{2}$ = 0.12, indicating that the IOR effect was larger when context was the same [−20 ms; *F*(1,118) = 178.76, *p* < 0.0001, $\eta_{p}^{2}$ = 0.60] than when it was different [−16 ms; *F*(1,118) = 112.25, *p* < 0.0001, $\eta_{p}^{2}$ = 0.49]. While the SOA main effect was significant [*F*(1,59) = 29.72, *p* < 0.0001, $\eta_{p}^{2}$ = 0.33], showing that responses were faster when the SOAs were the same, the interactions between SOA and other variables were not significant (*p*s > 0.20). Finally, analysis of error rates revealed no significant effects, *p*s > 0.15.

#### Analysis of the Cuing Effect as a Function of RT

**Figure 4:**
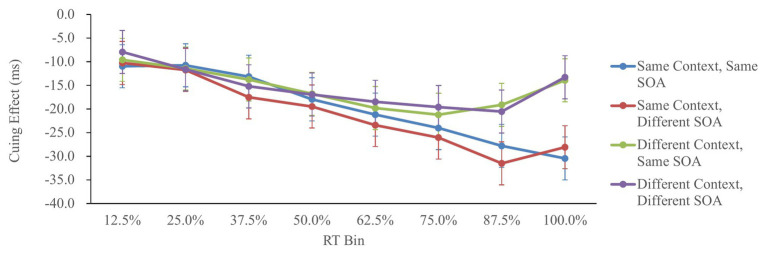
Cuing effect in Experiment 2, as a function of context, SOA, and RT bin. Cuing effect = invalid RT − valid RT. Error bars represent the 95% confidence interval, based on the method of Jarmasz and Hollands (2009).

Figure 4 shows the cuing effect in each condition as a function of the RT.

The cuing effects were analyzed by a 2 (context: same/different) × 2 (SOA: same/different) × 8 (RT bin: 12.5/25/37.5/50/62.5/75/87.5/100%) repeated-measures ANOVA. The main effect of context was significant, *F*(1,59) = 9.06, *p* < 0.01, $\eta_{p}^{2}$ = 0.13, showing larger IOR in the same-context condition. The main effect of RT bin was also significant, *F*(7,413) = 17.84, *p* < 0.01, $\eta_{p}^{2}$ = 0.23, showing that the IOR effects in the 50, 75, 87.5, and 100% RT bins were larger than that in the 12.5 and 25% RT bins; the IOR effects in the 75, 87.5, and 100% RT bins were larger than that in the 37.5% RT bin; and the IOR effect in the 87.5% RT bin was larger than that in the 50% RT bin (Tukey’s test, *p* < 0.05). More importantly, the interaction between context and RT bin was significant, *F*(7,413) = 3.43, *p* < 0.01, $\eta_{p}^{2}$ = 0.05, suggesting that the context modulated the IOR effect in the 87.5 and 100% RT bins [*F*(1,472) = 9.76, *p* < 0.01, $\eta_{p}^{2}$ = 0.02 and *F*(1,472) = 24.84 *p* < 0.01, $\eta_{p}^{2}$ = 0.05, respectively], but not in the 12.5, 25, 37.5, 50, 62.5, and 75% RT bins (*p* > 0.10). Other effects involving SOA were not significant, *p* > 0.20.

### Discussion

The design of Experiment 2 was identical to that of Experiment 1 except for further manipulation of the SOA between the fixation cue and the target. The results replicated the findings of Experiment 1. The effect of IOR was larger when the context was the same than when the context was different, thereby supporting the involvement of context-dependent memory retrieval in IOR.

Moreover, the analysis of the IOR effect as a function of RT bins provides evidence for time-dependent memory in IOR, similar to the findings in NP (Tse et al., 2011). That is, memory retrieval of the previous episode *via* context requires time to operate. When the RTs are longer, the previous episode is more likely to be retrieved by the context, and it is more likely that the impact of context on IOR will be observed.

In contrast to the findings of Experiment 2, it should be noted that the interaction between context and RT bins did not reach significance in Experiment 1. This may be related to the small effect size of this interaction ($\eta_{p}^{2}$ = 0.05). In addition, considering that the SOA between the fixation cue and the target was varied in Experiment 2 but not in Experiment 1, it is also possible that the interaction between context and RT bins is more likely to manifest when there is temporal uncertainty regarding the onset time of the target. For instance, it has been shown that temporal predictability modulated IOR in a localization task (Mondor, 1999) and in a discrimination task (Gabay and Henik, 2010), but not in a detection task (Gabay and Henik, 2008). In the present study, the contrast between Experiment 1 (higher temporal predictability) and Experiment 2 (lower temporal predictability) might be related to the effect of temporal predictability, although a detection task was used. Further studies are required to examine these speculations and to reveal the underlying mechanisms.

In Experiment 2, SOA similarity had no significant effect on IOR. One possible explanation is the relative salience of the contextual cues. Because the color context (red vs. green) is more salient than the SOA context (500 vs. 700 ms) in the present experiment, the color contextual cues are more likely to be encoded into memory representations and/or more likely to be used as retrieval cues (e.g., Eich, 1980). Hence, the color context had larger effects than the SOA context did.

Regarding the effect of color-based IOR, the main effect of color repetition was not significant. A closer look at the interaction between color repetition (i.e., context) and cue revealed that color repetition led to a cost in the valid condition [−4 ms; *F*(1,118) = 9.81, *p* < 0.01, $\eta_{p}^{2}$ = 0.08] and a nonsignificant benefit in the invalid condition [1 ms; *F*(1,118) = 0.49, *p* > 0.20, $\eta_{p}^{2}$ = 0.00]. In general, the phenomenon of color-based IOR is not reliable but may be observed when the stimuli are presented at the same location.

## General Discussion

The present study investigated the impact of contextual similarity between two successive targets on IOR in the target-target paradigm by manipulating the target color to create same and different contexts (i.e., the target color may be repeated or not). In Experiment 1, the SOA between the fixation cue and target was fixed. In Experiment 2, the SOA was varied to reduce preparatory responses to the target. Considering that Kwak and Egeth (1992) also manipulated location repetition and color repetition but did not observe reliable interaction between location repetition and color repetition, a neutral distractor was included at the beginning of each trial, as Law et al. (1995) suggested that the presence of neutral distractors is important for the manifestation of color-based IOR. Both experiments of the present study showed that IOR was larger in the same-context condition, supporting the role of context-dependent memory retrieval in IOR. According to Tipper et al. (2003), transient attentional states are stored in memory representations. Retrieval of these representations can lead to the reinstatement of transient attentional states. In other words, if a spatial location is associated with attentional inhibition and if this association is maintained in memory representations, retrieving these representations can produce an inhibitory effect at the associated spatial location. Because memory episodes are more likely to be retrieved when the context is the same (e.g., Tulving and Thomson, 1973), IOR is larger in the same – rather than different – context condition. In other words, the present study implies that memory retrieval plays an important role in IOR. Considering that studies on the role of memory retrieval in IOR is limited (a recent related study is the effect of retrieval of previous response on IOR; Hilchey et al., 2020), this highlights the importance of the present study and the need for further research.

The results of the present study can also be incorporated into other accounts and models of IOR. According to the detection cost account (Lupiáñez et al., 2007, 2013; Lupiáñez, 2010; Martín-Arévalo et al., 2013), exogenous cues interfere with attention capture by the target at the cued location. Target onset is usually able to capture attention. However, when the target is presented at a cued location, it is not spatially distinct from the onset of an exogenous cue at the same location. Consequently, target onset at the cued location is less capable of capturing attention. In the present study, the target at the cued location in the same-context condition is not distinct in its spatial location, shape, and color from the cue (target in the previous trial). By contrast, the target at the cued location in the different-context condition is not distinct in its spatial location and shape, but is distinct in color. Therefore, the IOR effect is larger in the same-context condition. Incorporating the results of the present study into the detection cost account of IOR suggests that the distinctiveness of target onset can be manipulated by varying the features between two successive targets in the target-target paradigm to further investigate the impact of target onset distinctiveness on target detection.

The results of the present study are consistent with a habituation account of IOR (Dukewich, 2009), with one modification. According to this account, IOR reflects habituation of orienting responses to the cued location. The original habituation account only emphasizes spatial habituation. The habituation account can explain the results of the present study if the concept of habituation is expanded to include habituation of nonspatial features.

The results of the present study are incompatible with the constructive retrieval account of IOR (Milliken et al., 2000). According to the constructive retrieval account, IOR is related to the relative efficiency of integrating the current perceptual event (e.g., a target at the cued location) with previous memory episodes (e.g., a cue at the cued location) and attentional capture by novel events (e.g., a target at the uncued location). When episodic integration at the old, cued location is less efficient than attentional capture at the novel, uncued location, responses to targets will be slower at the cued vs. uncued location, resulting in IOR. In the present study, a target-target paradigm was used, and the targets were identical on two successive trials in the cued, same-context condition. The constructive retrieval account predicts that this similarity between two successive events would facilitate episodic integration and hence reduce IOR. Thus, observing larger IOR in the same-context condition in the present study is inconsistent with the constructive retrieval account. Nevertheless, there may be multiple sources of IOR. They include retrieval of the inhibitory effect (Tipper et al., 2003), detection cost (Lupiáñez et al., 2007, 2013; Lupiáñez, 2010; Martín-Arévalo et al., 2013), and habituation (Dukewich, 2009), which produce larger IOR when the context is reinstated. Another is constructive retrieval (Milliken et al., 2000), which produces larger IOR when the context is altered. Further work is required to disentangle these sources of IOR.

Another important finding of the present study is the dependence of the contextual similarity effect on longer RTs. In the literature of NP, it has been demonstrated that the contextual similarity effect requires time to develop (e.g., Tse et al., 2011). That is, it is easier to observe the contextual similarity effect with slower rather than quicker responses. Following Tse et al.’s method, we also found that the contextual similarity effect in IOR was also more likely to manifest when the responses were long. Such a finding implies that while context-dependent memory retrieval plays a role in IOR, its effect is larger when there is sufficient time for context-dependent memory retrieval to take place. It should be noted that, in the present study, IOR occurred even when the RTs were short and when the context was different. This may further imply that IOR may involve multiple mechanisms: one manifests quickly and does not require context-based memory retrieval, while another requires time-dependent, context-dependent memory retrieval. We speculate that the latter may share a mechanism similar to that of long-term IOR (Tipper et al., 2003; Kessler and Tipper, 2004; Grison et al., 2005; Wilson et al., 2006). Further studies regarding the impact of context on long-term IOR and the time course of memory retrieval in long-term IOR are required to elucidate the possible mechanisms.

The findings that spatial IOR was modulated by color context might be interpreted from a different viewpoint: there was an effect of color-based IOR that was modulated by spatial context. Law et al. (1995) demonstrated that there was a repetition cost when the color of a to-be-detected color patch matched the color of a previous color patch. In addition, Kwak and Egeth’s (1992) Experiment 2 demonstrated that when both location repetition and color repetition were manipulated, color-based IOR occurred only at the cued location. Hu et al. (2011) also found color-based IOR at the cued location when complex displays were used. However, it should be noted that color-based IOR was not reliable in the present study. In Experiment 1, there was no evidence of color-based IOR (instead, there was a color-repetition benefit at the invalid location). In Experiment 2, a small (−4 ms) but significant color-based IOR was observed at the valid location. Considering all the findings of the present study, although a neutral distractor (Law et al., 1995) was included, consistent with the conclusion of Kwak and Egeth (1992), color-based IOR appears to be a less reliable phenomenon. Hence, the findings of the present study appear to be less relevant to the interaction between color-based IOR and spatial locations. Nevertheless, considering that a reliable interaction between location repetition and color repetition was observed across two experiments, it is plausible that instead of focusing on whether there is a location-based IOR and/or a color-based IOR, we should consider there to be a binding of color, spatial location, and attentional/processing/responding history according to the idea of event files (Hommel, 2004). Moreover, the integrated features can serve as cues for retrieving the event. Further studies of the interaction between location repetition, feature repetition, and processing/response repetition are required to test this possibility, such as the recent studies of memory retrieval of the previous response (e.g., Hilchey et al., 2018, 2020).

Finally, it should be noted that the present study can be further extended in several directions. First, the role of time course in context-dependent memory retrieval was evaluated by analyzing the vincentized cumulative RT distribution (Ratcliff, 1979) in the present study, rather than manipulating the time course. In a future study, the impact of time course can be further investigated by experimentally manipulating the response deadline (see Rosenstreich and Goshen-Gottstein, 2015, for a detailed discussion) and examining the contextual similarity effect in the fast vs. slow conditions. Second, recent studies suggest that attention may select the stimuli in a periodic manner (e.g., VanRullen et al., 2007; Landau and Fries, 2012), such as switching between the cue and target in a rhythmic manner (e.g., Chen et al., 2017). It remains a puzzle whether the IOR effect observed in the present study also involves an oscillatory mechanism, and this can be further studied by manipulating the SOA between the first trial and the second trial, similar to the study of Chen et al. (2017).

## Data Availability Statement

The datasets generated for this study are available on request to the corresponding author.

## Ethics Statement

The studies involving human participants were reviewed and approved by the Research Ethics Office of National Taiwan University. The patients/participants provided their written informed consent to participate in this study.

## Author Contributions

H-FC designed the study and drafted the manuscript. C-YK, MSC, and F-SH collected and analyzed the data. All authors contributed to the article and approved the submitted version.

### Conflict of Interest

The authors declare that the research was conducted in the absence of any commercial or financial relationships that could be construed as a potential conflict of interest.
